# Effects of GLP-1 receptor agonists on arrhythmias and its subtypes in patients with type 2 diabetes: A systematic review and meta-analysis

**DOI:** 10.3389/fendo.2022.910256

**Published:** 2022-08-11

**Authors:** Jinjing Wei, Ruxin Wang, Haowen Ye, Ying Wang, Lihong Wang, Xiaofang Zhang

**Affiliations:** ^1^ Department of Endocrinology and Metabolism, First Affiliated Hospital of Jinan University, Guangzhou, China; ^2^ Department Clinical Experimental Center, First Affiliated Hospital of Jinan University, Guangzhou, China

**Keywords:** Arrhythmia, Glucagon-like peptide 1 receptor agonist, T2DM, Meta-analysis, Systematic Reviews

## Abstract

**Purpose:**

An update of a systematic review and meta-analysis of the risk of arrhythmias and their subtypes in type 2 diabetic patients receiving glucagon-like peptide 1 receptor agonist (GLP-1RA) medication according to data from the Cardiovascular Outcome Trial(CVOT).

**Methods:**

Randomized controlled trials (RCT) on GLP-1RA therapy and cardiovascular outcomes in type 2 diabetes mellitus patients published in full-text journal databases such as MEDLINE (via PubMed), Embase, Clinical Trials.gov, and the Cochrane Library from establishment to March 1, 2022 were searched. We assessed the quality of individual studies by the Cochrane risk-of-bias algorithm. RevMan 5.4.1 software was use for calculating meta-analysis.

**Results:**

A total of 60,081 randomized participants were included in the data of these 8 GLP-1RA cardiovascular outcomes trials. Pooled analysis reported no significant effect on total arrhythmia [RR=0.96, 95% CI (0.96, 1.05), *p* =0.36], and its subtypes such as atrial fibrillation [RR=0.96, 95% CI (0.86, 1.07), *p* =0.43], atrial flutter [RR= 0.82, 95% CI (0.57, 1.19), *p* =0.30], atrial tachycardia [RR=0.64, 95% CI (0.20, 2.01), *p* =0.44)], sinoatrial node dysfunction [RR=0.74, 95% CI (0.44, 1.25), *p* =0.26], ventricular preterm systole [RR=1.42, 95% CI (0.62, 3.26), *p* =0.41], second degree AV block [RR=0.96, 95% CI (0.53, 1.72), *p* =0.88], complete AV block [RR=0.75, 95% CI (0.49, 1.17), *p* =0.21], ventricular fibrillation [RR=1.00, 95% CI (0.50, 2.02), *p* =1.00], ventricular tachycardia [RR=1.37, 95% CI (0.91, 2.08), *p* =0.13] from treatment with GLP-1RA versus placebo. However, the risk of hypoglycemia was reduced by about 30% [RR=0.70, 95% CI (0.57, 0.87), *p*=0.001] and the risk of pneumonia by about 25% [RR=0.85, 95% CI (0.75, 0.97), *p*=0.01], both statistically significant differences.

**Conclusion:**

In type 2 diabetic patients, treatment with GLP-1RA has no significant effect on the risk of major arrhythmias but significantly reduces the risk of hypoglycemia and pneumonia.

## 1 Introduction

Diabetes is one of the most common chronic diseases worldwide, and cardiovascular events are the most major complications in type 2 diabetics, including atrial and ventricular arrhythmias (1, 2). The prerequisite conditions of atherosclerosis present in type 2 diabetic patients due to inflammation, oxidative stress, glucotoxicity and other mechanisms persist or act together to cause most of these cardiovascular events. Some studies claim that the main risk factor for the development of atrial fibrillation in diabetic patients is not hyperglycemia, but the metabolic syndrome (one important feature of which is increased sympathetic activity) (1).

GLP-1RA is a new type of glucose-lowering drug that lowers HbA1c, modestly improves blood lipids and body weight, and decreases the risk of hypoglycemia by stimulating insulin secretion and lowering glucagon secretion, delaying gastric emptying, and reducing appetite (3). It has been observed that GLP-1R expression is present in human carotid body chemoreceptor cells and that GLP-1RA use decreases basal carotid body secretions and attenuates chemoreflex-induced sympathetic responses. The associated sympathetic overactive pattern induced by hyperglycemia can be abolished by the effect of GLP-1R activated on the carotid body (4). Extensive research data are now available to show that GLP-1RA has shown impressive positive effects in further reducing primary cardiovascular adverse events such as CV mortality (13% reduction), non-fatal stroke (16% reduction) and non-fatal myocardial infarction (9% reduction, although not at significant levels) by, among other things, reducing atherosclerosis (5).

The key role of the cardiac ANS in the development of arrhythmias has been confirmed by research that began as early as 60 years ago (6). The autonomic nervous system plays an influential role in the regulation of cardiac electrophysiology and arrhythmias, and the specific mechanisms differ among the types of arrhythmias. For example, in atrial fibrillation, sympathetic and parasympathetic activation is the most frequent trigger mechanism (7). From the above, it can be seen that diabetic patients can have a certain risk of arrhythmia, and the occurrence of arrhythmia has an uncuttable relationship with sympathetic nerves, and GLP-1RA can attenuate sympathetic nerve activity, so can GLP-1RA reduce the risk of arrhythmia in DM patients? A 2016 meta-analysis showed that exenatide was associated with an increased risk of arrhythmias in type 2 diabetes (OR 2.83; 95% CI: 1.06-7.57) (8). In 2021 another linked meta-analysis showed that in type 2 diabetic patients, using GLP-1RA treatment did not significantly affect the risk of major arrhythmia (9). Thus, it is clear that the controversial findings of before-and-after studies for arrhythmias and the few studies investigating the effect of GLP-1RA on the outcome of different arrhythmia subtypes, together with the recent publication of new RCTs on GLP-1RA on CVD outcome. Therefore, a new meta-analysis study is warranted.

According to the World Health Organization, Covid-19 disease that has emerged since the first case in December 2019 has resulted in up to 510 million confirmed cases, including 6.22 million deaths. Cases’ severity varied among all age populations depending on their comorbidities. Diabetes mellitus was found to be one of important risk factors which may contribute to the development of severe form of Covid-19, causing further inflammation and immune dysfunction that leads to the formation of cytokines storm which is life threatening (10–12). In addition to stimulating postprandial insulin secretion, GLP1-RA also seems to have beneficial properties such anti-inflammatory, anti-obesogenic, pulmonary protective effects and gut microbiome modulating effects (13). Recently the use of GLP1-RA, as one alternative to treat DM patients, had shown promising effect to reduce excessive inflammation-induced acute lung injury and improving Covid-19 outcome (14). Furthermore, an experimental study on rats showed that the use of GLP1-RA (liraglutide) was capable to stimulate pulmonary ACE2 expression, an enzyme that has been demonstrated to oppose the pathway that is responsible for the progression of acute respiratory distress syndrome (ARDS), including the one caused by SARS-CoV-2 infection (15, 16). Data from a meta-analysis in 2021 suggested that pre-admission use of GLP-1RA was associated with reduction in mortality rate from Covid-19 in patients with diabetes mellitus (OR 0.53; 95 %CI: 0.43–0.66, *p* < 0.00001, *I* ^2^ = 0%, random-effect modelling) (17). On this occasion, we propose to collect the occurrence of pneumonia as a secondary outcome to do a validation of interest.

Therefore, the intention of our analyses was to explore the impact of GLP-1RA treatment on the occurrence of several different types of arrhythmias in type 2 diabetic patients based on data extracted from relevant cardiovascular outcome trials, with the addition of some secondary indicators of interest, such as hypoglycemia and pneumonia cases.

## 2 Materials and methods

### 2.1 Search strategy

The English and Chinese literature on GLP-1RA associated with cardiovascular outcomes published since the establishment of the database until March 1, 2022 was searched manually by computer in MEDLINE (via PubMed), Embase, Clinical Trials.gov, and Cochrane Library databases. Search terms: “Glucagon-like peptide-1 receptor agonist”, “Cardiovascular”, “ cardiac failure”, “lixisenatide”, “exenatide”, “liraglutide “, “Semaglutide”, “Albiglutide”, “Dulaglutide”, “Efpeglenatide”, “placebo”, “Clinical Trials”.

### 2.2 Inclusion criteria and exclusion criteria

Inclusion criteria: (1) Randomized, double-blind, parallel-group, multicenter study of clinical trials; (2) Patients with type 2 diabetes who are at high cardiovascular risk (including but not limited to obesity, metabolic syndrome, insulin resistance, hypertension, dyslipidemia, etc.) are the primary study subjects; (3) Intervention with GLP-1 receptor agonist and control with placebo; (4) Data on arrhythmias, hypoglycemia, and pneumonia must be available in all trials for adverse events; (4) A more complete table of baseline patient characteristics is available; (5) Published English literature up to March 1, 2022.

Exclusion criteria: (1) Reviews, reports, and conference proceedings on GLP-1RA and cardiac arrhythmias; (2) Studies with inaccessible full text or incomplete data; (3) Repeatedly published or repeatedly included studies or studies with similar information; (4) Clinical trials that included type 1 diabetic patients.

### 2.3 Main and secondary results

#### 2.3.1 Primary outcome

Total arrhythmias and major arrhythmia types (including atrial fibrillation, atrial flutter, ventricular fibrillation, ventricular tachycardia, atrial tachycardia, sinus node dysfunction, ventricular anterior contraction, second degree AV block and complete AV block).

#### 2.3.2 Secondary outcomes

hypoglycemia (defined as diabetes mellitus in patients receiving medication as long as their blood glucose is below 3.9 mmol/L) and pneumonia (defined as pneumonia as an inflammation of the lungs with lobar or interstitial infiltrates).

### 2.4 Literature screening, data extraction and quality evaluation

Randomized controlled trials comparing GLP-1RA with placebo in type 2 diabetic patients at high cardiovascular risk were included. Outcomes of interest included atrial fibrillation, atrial flutter, ventricular fibrillation, ventricular tachycardia, atrial tachycardia, sinus node dysfunction, ventricular asystole, second degree AV block and complete AV block, hypoglycemia, and pneumonia. First, titles and abstracts were screened to assess their potential eligibility for inclusion, and then full-text checks were applied to determine final eligibility. The following information was collected using a predefined data extraction form: study information (trial name, sample size, drug name), patient characteristics (age, gender, baseline status and preexisting CVD history comorbidities), therapy information (regimen, dose) and outcome data (number of events per outcome). All outcomes of interest were dichotomous, first preferentially extracting data from ClinicalTrials.gov and secondarily selecting data from the original trial publication or secondary analysis of the same trial. The Cochrane Risk of Bias Tool was used to assess the quality of included studies (18). Bias was assessed in seven ways: selection bias (including random sequence generation, allocation concealment), implementation bias (whether subjects and trial personnel were blinded), measurement bias (whether outcome assessors were blinded), follow-up bias (whether outcome data were complete), reporting bias (whether study outcomes were selectively reported), and other bias (whether there were other sources of bias). The assessment criteria levels are classified as high, low or unclear. If one item was judged to be high, the overall risk of bias was judged to be high, and if all items were judged to be low, the risk of bias was judged to be low, otherwise it was unclear.

### 2.5 Statistical methods

Data were analyzed with RevMan 5.4.1, and effect analysis statistics were expressed as RR and 95% CI, *p <*0.05 being a statistically significant difference. Heterogeneity analysis among groups between studies was executed using the χ^2^ test, and the results are presented as *I^2^*. Fixed-effects models were used for analysis if there was homogeneity among studies (*p >*0.05 or *I^2^ ≤* 50%), and random-effects models were used for analysis if there was heterogeneity among studies (*p ≤* 0.05 and *I^2^* >50%). Sources of heterogeneity can be searched for by sensitivity analysis and subgroup analysis when large heterogeneity exists. Due to the number of trials being less than 10, publication bias was not evaluated.

## 3 Results

### 3.1 Procedure and outcomes of included literature

**Figure 1:**
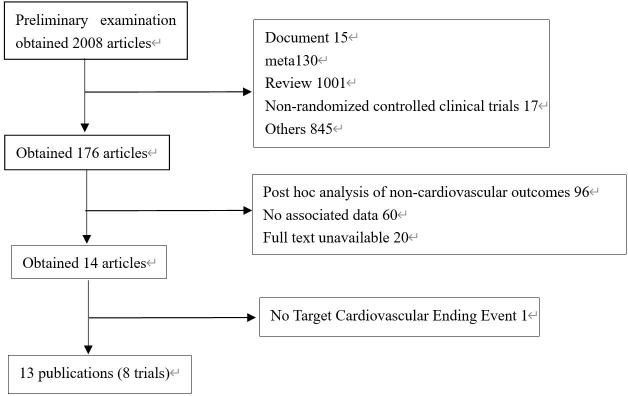
Process of studies’ selection.

2008 literatures were initially screened in the database according to keywords, and 176 were obtained after de-duplication and exclusion of review literature, irrelevant literature, incomplete data, unreported cardiovascular events, unspecified results, and incorrect study type, and then 8 clinical trials with a total of 68,001 patients were finally included after the screening process was repeated by two investigators again (**Figure 1**). The included reports, in chronological order, were ELIXA (19)、LEADER (20)、SUSTAIN-6 (21)、EXSCEL (22)、Harmony Outlets (23)、REWIND (24)、PIONEER 6 (25) and AMPLITUDE-O (26). Key trial and patient characteristics at baseline examination are shown in **Table 1**. All trials were of considerable size (>3000 patients). Of the 8 trials, ELIXA enrolled patients with a recent acute coronary syndrome event, and the study populations of the other 7 trials indicated in their inclusion criteria that they primarily included patients with stable cardiovascular disease or cardiovascular risk factors. In all eight trials, local investigators were encouraged to manage participants according to local guidelines.

**Table 1:** Baseline characteristics of the included studies.

Drug	ELIXA	LEADER	SUSTAIN-6	EXSCEL	Harmony Outcomes	REWIND	PIONEER 6	AMPLITUDE-O
	Lixisenatide	Liraglutide	Semaglutide	Exenatide	Albiglutide	Dulaglutide	Semaglutide	Efpeglenatide
Published Date	**2015.2**	**2015.12**	**2016.3**	**2017.4**	**2018.3**	**2018.8**	**2018.9**	**2020.12**
Number of people	**6068**	**9341**	**3297**	**14752**	**9463**	**9901**	**3183**	**4076**
Usage	**20μg sc qd**	**1.8mg sc qd**	**0.5 or 1mg sc qw**	**2mg sc qw**	**30 or 50mg qw**	**1.5mg sc qw**	**14mg po qd**	**4 or 6mg sc qw**
Median follow-up time(years)	**2.1**	**3.8**	**3.1**	**3.2**	**1.6**	**5.4**	**1.3**	**1.8**
Average age(years)	**60.3**	**64.3**	**64.6**	**61.9**	**64.1**	**66.2**	**66.0**	**64.5**
Male (n,%)	**4207(69.3%)**	**6003(64.3)**	**2002(60.1%)**	**9149(62%)**	**6569(69.4%)**	**5312(53.7%)**	**2176(68.4%)**	**2732(67%)**
BMI(kg/m^2^)	**30.2**	**32.5**	**32.8**	**31.8**	**32.3**	**32.3**	**32.3**	**32.7**
History of CVD (n,%)	**6068(100%)**	**7598(81%)**	**2735(83%)**	**10782(73%)**	**9463(100%)**	**3109(31.4%)**	**2695(84.7%)**	**3650(89.6%)**
Duration of DM disease	**9.2**	**12.8**	**13.9**	**13.1**	**14.2**	**10.5**	**14.9**	**15.4**
Mean HbA1c (%)	**7.7**	**8.7**	**8.7**	**8.1**	**8.7**	**7.3**	**8.2**	**8.9**
Heart failure (n, %)	**1358(22.4%)**	**1667(17.8%)**	**777(23.6%)**	**2389(16.2%)**	**1912(20.2%)**	**853(8.6%)**	**388(12.2%)**	**737(18.1%)**
Iusulin use(%)	**39**	**45**	**58**	**46**	**59**	**24**	**61**	**62**
Baseline eGFR(mL/min per1.73m^2^)	**76**	**<75**	**<75**	**76**	**79**	**77**	**74**	**72.4**
SGLT2I use(%)	**NA**	**NA**	**5(0.2)**	**77(0.9)**	**575(6.1)**	**3(0.0)**	**305(9.6%)**	**618(15.2%)**

Renal disease was defined as an estimated glomerular filtration rate (eGFR) of 25.0 to 59.9 ml per minute per 1.73 m ^2^ body surface area. eGFR was calculated using the four-variable formula for dietary adjustment for renal disease: 175 × (serum creatinine level [μmol/L]/88.4) - 1.154 × age (years) - 0.203 × 1.212 × 0.742.

### 3.2 Arrhythmia analysis

Separate data-analyses were estimated according to the type of adverse event arrhythmia disclosed, homogeneity of included studies was good for all events, with the following results:atrial fibrillation (*I ^2^ =*44%, *p* =0.08), atrial flutter (*I^2^=*0%, *p* =0.49), ventricular fibrillation (*I^2^=*0%, *p* =0.86), ventricular tachycardia (*I^2^=*0%, *p* =0.42), atrial tachycardia (*I^2^=*0%, *p* =0.45), sinus node (*I^2^=*0%, *p* =0.54), ventricular precontraction (*I^2^=*0%, *p* =0.64), second degree AV block (*I^2^=*0%, *p* =0.84), and complete AV block (*I^2^=*40%, *p* =0.13), so a fixed-effect model was used to combine effect sizes.

**Figure 2:**
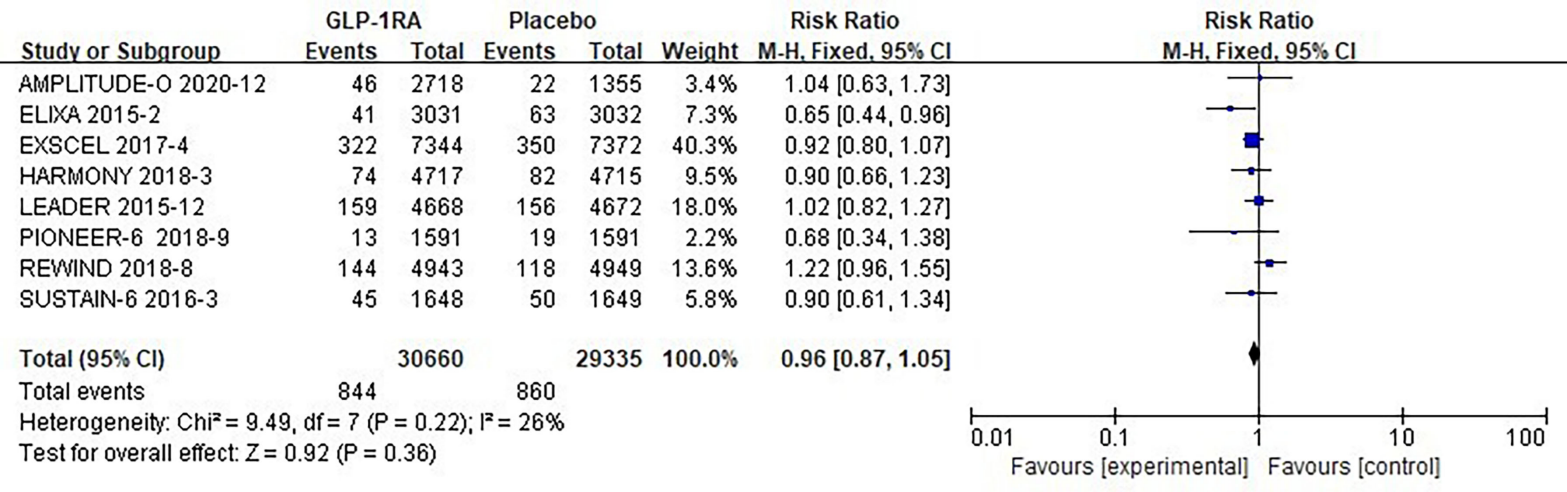
Forest plot of the risk of total arrhythmias.

The eight studies collected for mate-analysis of the effect of GLP-1RA on total arrhythmias are listed in **Figure 2**. No significant heterogeneity among studies (*I^2^=*26%, *p*=0.22), so the fixed-effects model was used to combine the effect sizes. Pooled analysis reported no significant effect on total arrhythmias outcomes from treatment with GLP-1RA versus placebo [RR = 0.96, 95% CI (0.87–1.05), *p* = 0.36].

#### 3.2.1 Atrial fibrillation

**Figure 3:**
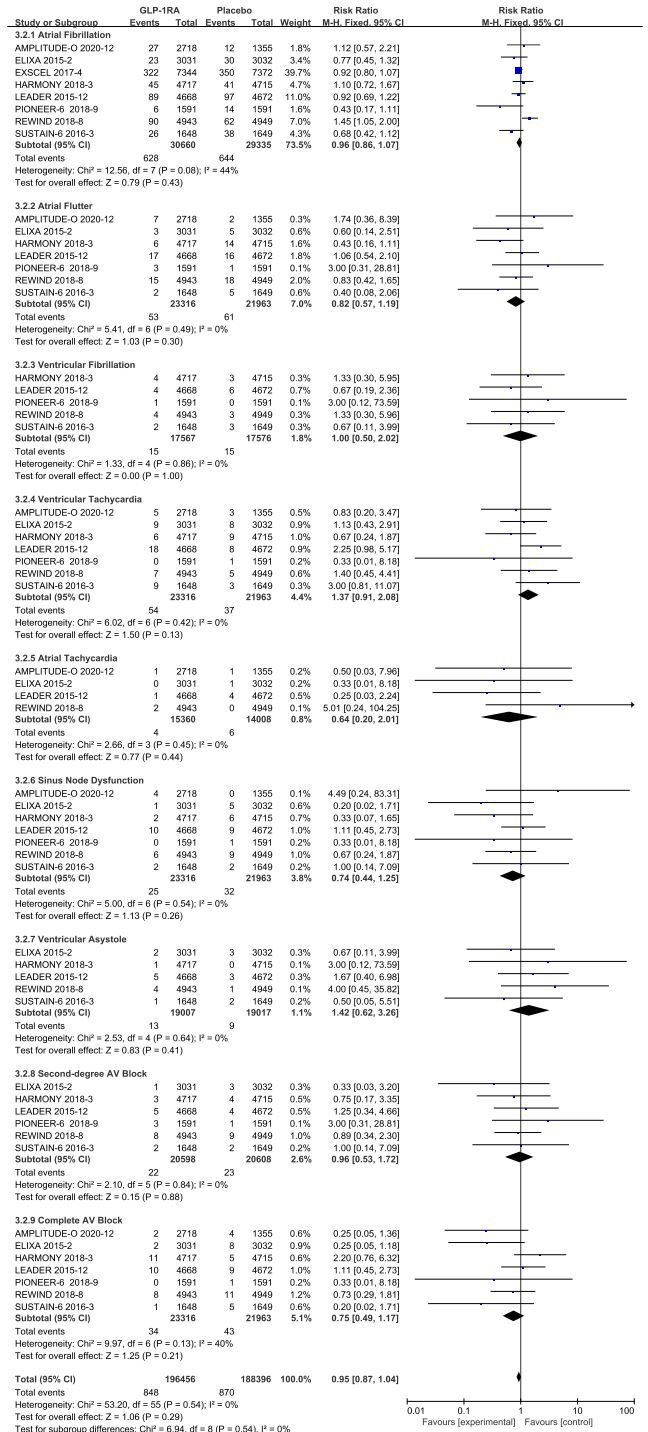
Forest plot of risk for each arrhythmia subtype.

The eight studies collected for mate-analysis of the effect of GLP-1RA on atrial fibrillation are listed in **Figure 3**. No significant heterogeneity among studies (*I^2^=*44%, *p*=0.08), so the fixed-effects model was used to combine the effect sizes. Pooled analysis reported no significant effect on atrial fibrillation outcomes from treatment with GLP-1RA versus placebo [RR=0.96, 95% CI (0.86, 1.07), *p* =0.43].

#### 3.2.2 Atrial flutter

The seven studies collected for mate-analysis of the effect of GLP-1RA on atrial flutter are listed in **Figure 3**. No significant heterogeneity among studies (*I^2^=*0%, *p*=0.49), so the fixed-effects model was used to combine the effect sizes. Pooled analysis reported no significant effect on atrial flutter outcomes from treatment with GLP-1RA versus placebo [RR=0.82, 95% CI (0.57, 1.19), *p* =0.30].

#### 3.2.3 Ventricular fibrillation

The five studies collected for mate-analysis of the effect of GLP-1RA on ventricular fibrillation are listed in **Figure 3**. No significant heterogeneity among studies (*I^2^=*0%, *p*=0.86), so the fixed-effects model was used to combine the effect sizes. Pooled analysis reported no significant effect on ventricular fibrillation outcomes from treatment with GLP-1RA versus placebo [RR=1.00, 95% CI (0.50, 2.02), *p* =1.00].

#### 3.2.4 Ventricular tachycardia

The seven studies collected for mate-analysis of the effect of GLP-1RA on ventricular tachycardia are listed in **Figure 3**. No significant heterogeneity among studies (*I^2^=*0%, *p*=0.42), so the fixed-effects model was used to combine the effect sizes. Pooled analysis reported no significant effect on ventricular tachycardia outcomes from treatment with GLP-1RA versus placebo [RR=1.37, 95% CI (0.91, 2.08), *p* =0.13].

#### 3.2.5 Atrial tachycardia

The four studies collected for mate-analysis of the effect of GLP-1RA on atrial tachycardia are listed in **Figure 3**. No significant heterogeneity among studies (*I^2^=*0%, *p*=0.45), so the fixed-effects model was used to combine the effect sizes. Pooled analysis reported no significant effect on atrial tachycardia outcomes from treatment with GLP-1RA versus placebo [RR=0.64, 95% CI (0.20, 2.01), *p* =0.44].

#### 3.2.6 Sinus node dysfunction

The seven studies collected for mate-analysis of the effect of GLP-1RA on Sinus Node Dysfunction are listed in **Figure 3**. No significant heterogeneity among studies (*I^2^=*0%, *p* =0.54), so the fixed-effects model was used to combine the effect sizes. Pooled analysis reported no significant effect on Sinus Node Dysfunction outcomes from treatment with GLP-1RA versus placebo [RR= 0.74, 95% CI (0.44, 1.25), *p* =0.26].

#### 3.2.7 Ventricular asystole

The six studies collected for mate-analysis of the effect of GLP-1RA on ventricular asystole are listed in **Figure 3**. No significant heterogeneity among studies (*I^2^=*0%, *p* =0.64), so the fixed-effects model was used to combine the effect sizes. Pooled analysis reported no significant effect on ventricular asystole outcomes from treatment with GLP-1RA versus placebo [RR= 1.42, 95% CI (0.62, 3.26), *p* =0.41].

#### 3.2.8 Second-degree AV block

The six studies collected for mate-analysis of the effect of GLP-1RA on second-degree AV block are listed in **Figure 3**. No significant heterogeneity among studies (*I^2^=*0%, *p* =0.84), so the fixed-effects model was used to combine the effect sizes. Pooled analysis reported no significant effect on second-degree AV block outcomes from treatment with GLP-1RA versus placebo [RR =0.96, 95% CI (0.53, 1.72), *p* =0.88].

#### 3.2.9 Complete AV block

The seven studies collected for mate-analysis of the effect of GLP-1RA on complete AV block are listed in **Figure 3**. No significant heterogeneity among studies (*I^2^=*40%, *p* =0.13), so the fixed-effects model was used to combine the effect sizes. Pooled analysis reported no significant effect on complete AV block outcomes from treatment with GLP-1RA versus placebo [RR=0.75, 95% CI (0.49, 1.17), *p*=0.21].

### 3.3 Analysis of secondary outcomes

#### 3.3.1 Hypoglycemic risk analysis

**Figure 4:**
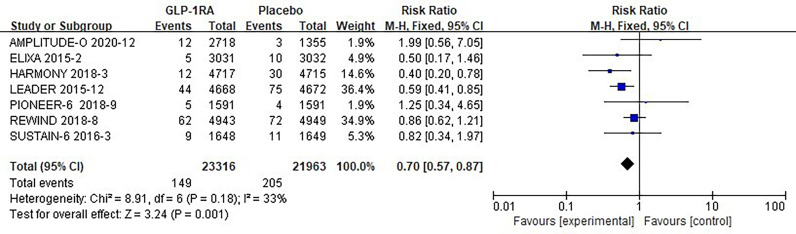
Forest plot of the risk of hypoglycemia.

The seven studies collected for mate-analysis of the effect of GLP-1RA on hypoglycemia are listed in **Figure 4**. No significant heterogeneity among studies (*I^2^=*33%, *p* =0.18), so the fixed-effects model was used to combine the effect sizes. Pooled analysis reported statistically significant effect on hypoglycemia outcomes from treatment with GLP-1RA versus placebo [RR=0.70, 95% CI (0.57, 0.87), *p*=0.001], showed that the risk of hypoglycemia was about 30% lower in the GLP-1RA treatment group than the placebo group.

#### 3.3.2 Pneumonia risk analysis

**Figure 5:**
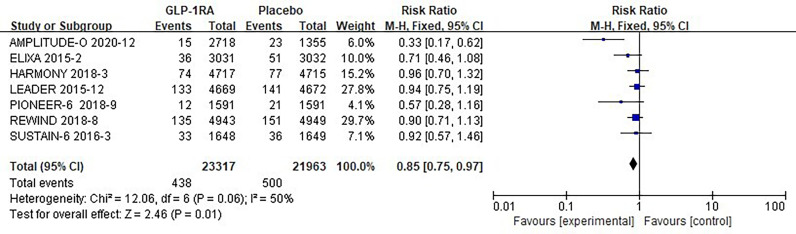
Forest plot of the risk of pneumonia.

The seven studies collected for mate-analysis of the effect of GLP-1RA on pneumonia are listed in **Figure 5**. No significant heterogeneity among studies (*I^2^=*50%, *p* =0.06), so the fixed-effects model was used to combine the effect sizes. Pooled analysis reported statistically significant effect on hypoglycemia outcomes from treatment with GLP-1RA versus placebo [RR=0.85, 95% CI (0.75, 0.97), *p*=0.01], showed that the risk of pneumonia was approximately 25% lower in the GLP-1RA treatment group than in the placebo group.

### 3.5 Overall analysis

A total of 60,081 randomized participants were included in the data of these 8 GLP-1RA cardiovascular outcomes trials. Pooled analysis reported no significant effect on not only total arrhythmia [RR=0.96, 95% CI (0.96, 1.05), *p* =0.36] (**Figure 2**), but also its subtypes atrial fibrillation [RR=0.96, 95% CI (0.86, 1.07), *p* =0.43] (**Figure 3**), atrial flutter [RR= 0.82, 95% CI (0.57, 1.19), *p* =0.30] (**Figure 3**), atrial tachycardia [RR=0.64, 95% CI (0.20, 2.01), *p* =0.44)] (**Figure 3**), sinoatrial node dysfunction [RR=0.74, 95% CI (0.44, 1.25), *p* =0.26] (**Figure 3**), ventricular preterm systole [RR=1.42, 95% CI (0.62, 3.26), *p* =0.41] (**Figure 3**), second degree AV block [RR=0.96, 95% CI (0.53, 1.72), *p* =0.88] (**Figure 3**), complete AV block [RR=0.75, 95% CI (0.49, 1.17), *p* =0.21] (**Figure 3**), ventricular fibrillation [RR=1.00, 95% CI (0.50, 2.02), *p* =1.00] (**Figure 3**), ventricular tachycardia [RR=1.37, 95% CI (0.91, 2.08), *p* =0.13] (**Figure 3**) from treatment with GLP-1RA versus placebo. However, there was a statistically significant reduction in the risk of hypoglycemia by about 30% [RR=0.70, 95% CI (0.57, 0.87), *p* =0.001] (**Figure 4**), and pneumonia by about 25% [RR=0.85, 95% CI (0.75, 0.97), *p* =0.01] (**Figure 5**).

## 4 Discussion

Rarely has the occurrence of arrhythmias in patients with type 2 diabetes been studied in clinical research. A 2021 meta-analysis only briefly summarized the results, without in-depth discussion and systematic elaboration, coupled with the fact that there were new clinical trials that were not included. The risks of arrhythmias and their subtypes in published high-quality CVOTs of GLP-1RA drug therapy in patients with type 2 diabetes up to March 1, 2022, were therefore extracted, based on this, we also conducted a systematic review and meta-analysis. The novelty of this study is that we included the most recent AMPLITUDE-O trial, which were not included in the 2021 meta-analysis (9), and we also looked at the risk of hypoglycemia and pneumonia. We included a sample of 60,081 participants from 8 CVOT trials, of which 46,100 had a history of cardiovascular disease (76.7%), and showed that GLP-1RA had no effect on the risk of total arrhythmias [RR=0.96, 95% CI (0.96, 1.05), *p* =0.36] in patients with type 2 diabetes. Pooled data suggest that GLP-1RA treatment has no effect on the risk of various types of arrhythmias. The risk of hypoglycemia was statistically significantly reduced by approximately 30% [RR=0.70, 95%CI(0.57, 0.87),*p*=0.001] and pneumonia by approximately 25% [RR=0.85, 95%CI(0.75, 0.97), *p*=0.01]. Based on the differences in primary and secondary outcome results seen in our analysis, we speculate that the neutral effect of GLP-1RA on arrhythmias may be due to the pharmacological effects of GLP-1RA itself, but may also be related to a reduction in the risk of hypoglycemia and thus some decrease in the sympathetic excitatory effects of stressful events。The results of the Cochrane risk of bias assessment among the included studies were of high quality, with low publication bias and good homogeneity, so no further sensitivity analysis was required. Compared with other CVOTs involving GLP-1RA, participants in the AMPLITUDE-O trial had the longest duration of diabetes (15 years), the lowest mean eGFR (72 ml/min/1.73 m 2), a higher proportion of nephropathy (eGFR <60 mL/min) at baseline (32%), the highest glycated hemoglobin (8.9%), and the highest percentage of sodium -glucose transporter 2 inhibitor (SGLT-2i) use (15%), suggesting that the AMPLITUDE-O trial was in a population with poorer underlying conditions (26). This suggests that GLP-1RA does not significantly increase the risk of arrhythmia even in patients with the longest disease duration or the most comorbidities. The conclusions reached in the present study are inconsistent with the 2016 meta-analysis of arrhythmias involving 54,758 enterostatin users and 48,175 controls, probably because the 2016 study was an arm-to-arm subgroup analysis of all enterostatin classes, and the conclusions reached were relative within the subcategories of GLP-1RA or DPP-4 inhibitors, whereas our study did not perform further drug type analysis because there was no significant heterogeneity, and was a conclusion drawn from a comprehensive overall analysis.

In clinical practice, arrhythmias cannot avoid being associated with changes in heart rate. A study published in diabetes care in 1999 showed that continuous infusion of GLP-1 did not increase blood pressure and heart rate in patients with type 2 diabetes (27). Another study published in JCI in 2002 showed EXN-4 may have a dose-dependent increase in blood pressure and heart rate, both after central and peripheral dosing (28). A 2015 meta-analysis revealed an increase in heart rate compared to placebo of 3.35 (95% CI: 1.23-5.50), 2.06 (95% CI: 0.43, 3.74) and 2.35 (95% CI: 0.94-3.76) beats/min by exenatide (2 mg once a week), liraglutide (1.2 mg once a day) and liraglutide (1.8 mg once a day) (29). Results of a 2016 clinical trial conducted in 10 healthy overweight men (20-27 years old) showed that acute GLP-1RA exenatide administration induced moderate but consistent heart rate acceleration and increased SBP and sympathetic activity markers in healthy overweight men (30). The effect of GLP-1RA on heart rate was variable, with no detailed description of heart rate found in all eight CVOTs included. Lack of data on the origin of the patients limited our further subgroup analysis to some extent. The potential mechanism for GLP-1RA-associated HR acceleration in humans remains speculative. The results of numerous randomized controlled trials have shown that GLP-1 may directly enhance sympathetic activity or lower parasympathetic nervous system activity (31). In addition, induction of vasodilation by GLP-1 may initially lead to a reduction in total peripheral resistance, resulting in negative feedback regulation of reflex tachycardia (32). So far, it has been proposed several hypotheses to account for GLP-1RA-induced heart rate acceleration: 1) regulation of the autonomic nervous system balance in the heart, 2) a reflex tachycardia that is responsive to vasodilation and 3) immediate stimulation of GLP-1 receptors on the sinoatrial cells (30, 33). Another study found that GLP-1R expression was present in human carotid body chemoreceptor cells and that GLP-1RA use decreased carotid body ID basal secretion and attenuated chemoreflex-induced sympathetic responses. The associated sympathetic overactive pattern induced by hyperglycemia can be abolished by the effect of GLP-1R activated on the carotid body (4). Mechanisms by which GLP-1RA achieves cardiac benefit in the absence of significant arrhythmias are partially explained, pending further future experiments to verify this relationship.

The new crown pneumonia outbreak in late 2019 poses a significant threat to human health, and poor glycemic control (e.g. in those with a high frequency of hypoglycemia) may increase oxidative stress and promote innate or adaptive immune dysfunction, leading to an increased chance of infection (34). Therefore, it is important to control blood glucose levels as part of the treatment strategy for patients with COVID-19 (17). In addition to pancreas, GLP-1-based drugs show anti-inflammatory and immunoregulatory effect in multiple organs. GLP‐1R is abundantly expressed in multiple organs including brain, pancreas, kidney, stomach, heart, and predominantly in lung epithelia and immune cells (35). It is well known that acute respiratory distress syndrome represents the most severe form of COVID-19. The so-called ‘Cytokine Storm’ that happens in this syndrome is characterized by the highest levels of inflammatory cytokines which damage alveolar epithelial cells in the lung and inactivate pulmonary surfactant, resulting in the formation of the hyaline membrane and lung parenchyma breakdown. With this background, GLP-1RAs might provide an opportunity to break the remarkable inflammation process, exerting broad anti-inflammatory actions and reducing biomarkers of systemic inflammation in particular in human subjects with type 2 diabetes and people with obesity (36). GLP‐1RA mainly express its anti‐inflammatory properties through the suppression of cytokine and chemokine production, stimulation of eNOS/sGC/PKG signaling pathway, inactivation of the NF‐ĸB signaling, as well as attenuation of thioredoxin‐interacting protein levels (37). Preclinical studies showed that GLP-1RAs reduce cytokine production, attenuate pulmonary inflammation and preserve lung function in rats and mice with experimental lung injury (38, 39). Animal studies in mice with experimental lung injury also showed that GLP‐1RA reduce mucus secretion, and preserve lung function (40–44). In an animal study conducted at rats, GLP-1 improved pulmonary physiology and surfactant production (16). In addition, Rogliani et al. reported that GLP‐1RAs improves forced expiratory volume in 1 s ‐ FEV1, forced vital capacity ‐ FVC, and maximal expiratory flow at 75% and 50% ‐ MEF75 and MEF50 in diabetes mellitus patients, regardless on their blood glucose levels (45). GLP-1 owns anti-inflammatory and anti-atherogenic properties. Several inflammatory markers and cardiovascular markers (e.g. C-reactive protein) are reduced in Type 2 diabetes patients treated with GLP-1RA (46). GLP-1 inhibited IL-1β production, cytokine secretion, interfere nuclear factor-kB pathway, and promoted survival after lipopolysaccharide induced systemic inflammation in rats. The fact that GLP-1 displays anti-inflammatory effects and GLP-1RA presence in the lung further support that GLP-1RA might attenuate acute lung disease (35, 46, 47). Related studies have shown that GLP1-RA is an excellent drug for the treatment of non-severe COVID-19 (insulin remains the treatment of choice for severe COVID-19 combined with type 2 diabetes because it is highly effective in controlling blood glucose levels, has a low risk of hypoglycemia, and has no significant disadvantages that may interfere with the metabolism of patients with COVID-19 diabetes) (48). This coincides with our findings demonstrating that GLP-1RA does reduce the risk of pneumonia and has undeniable positive and safe effects in patients with novel coronavirus pneumonia. It is well known that acute respiratory distress syndrome represents the most severe form of COVID-19. The so-called ‘Cytokine Storm’ that happens in this syndrome is characterized by the highest levels of inflammatory cytokines which damage alveolar epithelial cells in the lung and inactivate pulmonary surfactant, resulting in the formation of the hyaline membrane and lung parenchyma breakdown. With this background, GLP-1RAs might provide an opportunity to break the remarkable inflammation process, exerting broad anti-inflammatory actions and reducing biomarkers of systemic inflammation in particular in human subjects with type 2 diabetes and people with obesity (36).

Despite a large body of scientific research, GLP-1RA demonstrated a positive effect with a 14% reduction in major cardiovascular events (MACE) [HR=0.86, 95%CI(0.79-0.94), *p*=0.006] and no significant heterogeneity was observed between subgroups of individuals with or without cardiovascular disease (*p*=0.127). It also reduced the risk of cardiovascular death by 13% (*p*=0.016), non-fatal stroke by 16% (*p*=0.007), hospitalization for heart failure by 10% (*p*=0.023), and all-cause mortality by 12% (*p*=0.012) (6). However, there appears to be a neutral effect on the risk of arrhythmia. Our findings suggest that GLP-1RA may has no effect on the risk of most types of arrhythmias compared with placebo. It demonstrates that despite the sympathetic excitatory effect of GLP-1RA, GLP-1RA exhibits a degree of safety in terms of the risk of arrhythmia in patients with type 2 diabetes, with the implication that the use of GLP-1RA analogues may be considered to improve or maintain glucose-lowering therapy in patients with a long history of disease when clinically encountering patients with arrhythmias that meet the indications but for whom the use of SGLT2i is contraindicated. From another perspective, our findings serve as reverse evidence that further positively supports the consistent results presented by numerous international studies so far: GLP-1RA treatment can indeed bring cardiovascular benefits and safety to diabetic patients.

Despite our comprehensive systematic evaluation of the RCTs of GLP-1RA, some limitations remain. First, none of the included trials were specifically designed to assess arrhythmic outcomes with GLP-1RA, and all trials were designed to assess the safety of GLP-1RA. Further validation could be done in future by designing large clinical studies with arrhythmia as an endpoint. Second, different chemical structures, pharmacological profiles, and modes of action of these GLP-1RA are available, and although the differences are subtle, head-to-head cardiovascular outcomes trials for this class of drugs are not available, which limits the ability to detect differences in outcomes between GLP-1 agonists of different structures or potencies and to rule out whether they affect treatment outcomes. Third, differences in trial populations including baseline data could also be a potential explanation for differences in study outcomes. Nevertheless, we only collected overall data in our analysis, which limited our capability to further explore any subgroups of interest, to group trials according to combinations of disease processes, such as SGLT2i with GLP-1RA, amount of atrial fibrillation events in patients with or without pre-existing heart failure, and understanding of missing data remains to be explored. Therefore, the findings of the subgroup analysis have to be interpreted cautiously and further investigations are needed to assess whether GLP-1RA may reduce the incidence of arrhythmias in patients with multiple comorbidities. Fourth, the follow-up time of the study was also one of the limitations. Although these studies are considered long-term randomized controlled trial studies, they still only cover roughly 2-4 years, and patients may have been using GLP-1RA in clinical practice for a very short period of time. It is still not possible to exclude additional benefits or damages that may arise from shorter or longer exposure times. Fifth, the prevalence of these combined treatment effect sizes is determined by the study population, which is characterized by low insulin secretory capacity, insulin resistance and mild obesity relative to the American group, and regional subgroup analysis may provide more informative information if necessary. Sixth, no animal or cell-based experiments were focused on in this study. The above limitations may weaken the persuasiveness of our study, but the majority of eligible studies selected by 2 different investigators based on strict inclusion criteria were of high quality through a comprehensive literature search of 4 databases. Therefore, we believe it is reasonable to draw conclusions from this meta-analysis.

## 5 Conclusion

Overall, our study showed a neutral effect of GLP-1RA on the risk of major arrhythmias. However, it exactly in turn support the favorable effect of GLP-1RA on cardiovascular outcomes in patients with type 2 diabetes. The secondary outcomes of hypoglycemia and pneumonia showed statistically significant risk reduction, which provides further rationale for the safety of GP-1RA treatment. However, additional randomized clinical trials are needed in the future to confirm this conclusion.

## Data availability statement

The raw data supporting the conclusions of this article will be made available by the authors, without undue reservation.

## Author contributions

JW designed the research, JW and RWcontributed to the literature database search, data collection, data extraction, data analysis, and writing of the manuscript. JW, RW, HY and YW participated in the discussion. XZ and LW reviewed and revised this article. All authors contributed to the article and approved the submitted version.

## Funding

This study was supported by Talent introduction funding project of the First Affiliated Hospital of Jinan University (no.808026) and Basic Scientific Research Project of central Universities of Jinan University (no.21622301).

## Conflict of interest

The authors declare that the research was conducted in the absence of any commercial or financial relationships that could be construed as a potential conflict of interest.

## Publisher’s note

All claims expressed in this article are solely those of the authors and do not necessarily represent those of their affiliated organizations, or those of the publisher, the editors and the reviewers. Any product that may be evaluated in this article, or claim that may be made by its manufacturer, is not guaranteed or endorsed by the publisher.
