# Supplementary material for: Effects of GLP-1 receptor agonists on arrhythmias and its subtypes in patients with type 2 diabetes: A systematic review and meta-analysis
Source: Front Endocrinol (Lausanne). 2022 Aug 11;13:910256. doi: 10.3389/fendo.2022.910256 (PMC9403613; doi:10.3389/fendo.2022.910256)

**Additional information**

**1、Literature screening and retrieval:**

((((((((((glucagon like peptide-1 receptor agonists) OR (GLP-1 receptor agonists)) OR (Lixisenatide)) OR (exenatide)) OR (liraglutide)) OR (Semaglutide)) OR (Albiglutide)) OR (Dulaglutide)) OR (Efpeglenatide)) ) AND (Cardiovascular[Title/Abstract]) AND ((ffrft[Filter]) AND (randomizedcontrolledtrial[Filter]) AND (data[Filter]))

**2、Figure ：Risk of bias graph**


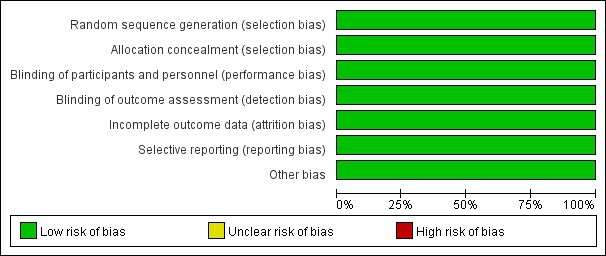

Supplement: Supplementary file 1 [file DataSheet_1.docx]
